# *FGFR4 *Gly^388^Arg polymorphism contributes to prostate cancer development and progression: A meta-analysis of 2618 cases and 2305 controls

**DOI:** 10.1186/1471-2407-11-84

**Published:** 2011-02-24

**Authors:** Bin Xu, Na Tong, Shu Q Chen, Li X Hua, Zeng J Wang, Zheng D Zhang, Ming Chen

**Affiliations:** 1 Department of Urology, The Affiliated ZhongDa Hospital of Southeast University, 87 Dingjia Bridge Hunan Road, Nanjing, 210009, China; 2 Department of Molecular and Genetic Toxicology, Cancer Center of Nanjing Medical University, 140 Hanzhong Road, Nanjing 210029, China; 3 Department of Urology, The First Affiliated Hospital of Nanjing Medical University, 300 Guangzhou Road, Nanjing, 210029, China

## Abstract

**Background:**

Fibroblast growth factor receptor 4 (FGFR4) displays multiple biological activities, including mitogenic and angiogenic activity, and plays important roles in the etiology and progression of prostate cancer. Gly^388^Arg polymorphism in *FGFR4 *gene has been reported to be involved in prostate cancer incidence and aggressiveness in several studies. To derive a more precise estimation of the relationship, a meta-analysis was performed.

**Methods:**

Odds ratios (ORs) with 95% confidence intervals (CIs) were estimated to assess the association.

**Results:**

The Arg^388 ^allele increased prostate cancer risk compared with Gly^388 ^allele (OR = 1.17, 95% CI = 1.07-1.29). When stratified by race, there was a significantly increased prostate cancer risk in Asian and Caucasian populations. Moreover, prostate cancer patients with Arg/Arg genotype had a 1.34-fold increased risk of advanced prostate cancer (95% CI: 1.03-1.74) compared with those with Gly/Gly+Gly/Arg genotype.

**Conclusion:**

This meta-analysis showed the evidence that *FGFR4 *Gly^388^Arg polymorphism was associated with an increased risk of prostate cancer development and progression, suggesting that *FGFR4 *Gly^388^Arg polymorphism could be a marker for prostate cancer development and progression.

## Background

Prostate cancer is the most frequently diagnosed solid tumor and the second leading cause of cancer-related death among American men, with an estimated 192,280 new cases and 27,360 deaths in the United States in 2009[1]. The etiology of human prostate cancer is complex and largely remains unknown.

Fibroblast growth factor receptor 4 (FGFR4) belongs to the family of fibroblast growth factor receptors (FGFR1-4), which display multiple biological activities, including mitogenic and angiogenic activity, with a consequent crucial role in cell differentiation, development, hormonal and proliferative signaling in response to more than 20 known ligands[2,3]. In light of its involvement in the regulation of essential biologic mechanisms, FGF signaling is also likely to play a role in tumor growth and progression; indeed, dysreglation of this pathway has been demonstrated in several tumor types[3]. Recently, FGFR4 was found to be more abundantly expressed in malignant than benign prostate cells and in vitro suppression of FGFR4 expression effectively blocked prostate cancer proliferation and invasion[4]. Moreover, strong expression of FGFR4 in prostate cancer cells, as assessed by immunohistochemistry, is significantly associated with increased clinical stage and tumor grade and decreased patient survival[5].

A germ line polymorphism in *FGFR4 *gene, resulting in different expression of FGFR4 containing either glycine (Gly^388^) or arginine (Arg^388^) at codon 388 in the transmembrane domain of the receptor was identified several years ago. In addition, the *FGFR4 *Arg^388 ^allele may predispose cancer patients to disease progression, based on the reported significant association between *FGFR4 *genotype and tumor aggressiveness or patients' survival in several cancers[6,7].

To date, several studies had been reported to focus on the association between this polymorphism and incidence and aggressiveness of prostate cancer[4,8-12]. However, a single study may be too underpowered to detect a possible small effect of the polymorphism on prostate cancer, especially when the sample size is relatively small. Hence, we carried out a meta-analysis of all eligible case-control studies to derive a more precise estimation of the association of *FGFR4 *Gly^388^Arg polymorphism with prostate cancer.

## Methods

### Publication search

PubMed and EMBASE were searched (the last search update on the 1^st ^Nov. 2010) using the search terms: '*FGFR4 *or fibroblast growth factor receptor 4', 'polymorphism', 'Gly^388^Arg or rs351855' and 'prostate cancer or prostate neoplasm'. All published English language papers with available full text matching the eligible criteria were retrieved. In addition, we checked all the references of relevant reviews and eligible articles that our search retrieved. Two investigators (BX and SQC) searched the literature and extracted data independently.

### Inclusion, exclusion criteria and data abstraction

For inclusion in the meta-analysis, the identified articles had to provide information on: (1) evaluation of *FGFR4 *Gly^388^Arg polymorphism and prostate cancer risk, (2) using a case-control design and (3) containing information about available genotype frequency that can help infer the results in the papers. Major reasons for the exclusion of studies were: (1) no control population; (2) no usable data reported; (3) duplicates. For each of the eligible case-control studies, the following data were collected: the first author's last name, year of publication, country of origin, ethnicity, numbers of genotyped cases and controls, genotyping methods.

### Statistical analysis

The strength of the association between the *FGFR4 *Gly^388^Arg polymorphism and prostate cancer risk was measured by ORs with 95% confidence intervals (CIs). We explored the association between allele Arg^388 ^and prostate cancer development and progression, as well as homozygote comparison (Arg/Arg vs. Gly/Gly), dominant genetic model [(Gly/Arg+Arg/Arg) vs. Gly/Gly] and recessive model [Arg/Arg vs. (Gly/Gly+ Gly/Arg)]. Heterogeneity assumption was checked by a chi-square-based *Q*-test[13]. A *P*-value of more than 0.05 for the *Q*-test indicated a lack of heterogeneity among the studies, so the summary OR estimate of each study was calculated by the fixed-effects model (the Mantel-Haenszel method). Otherwise, the random effects model (DerSimonian and Laird method) was used[14,15]. The significance of the pooled OR was determined by the *Z*-test, and *P *< 0.05 was considered as statistically significant. To evaluate the ethnic-specific effect, subgroup analysis was conducted on the basis of different ethnicities.

Evidence of publication bias was determined using Begg's[16] and Egger's[17] formal statistical test and by visual inspection of the funnel plot. All statistical analyses were performed with Stata software (version 10.0; StataCorp LP, College Station, TX), using two-sided *P *values.

## Results

### Study characteristics

Using the searching terms, seven papers were reviewed in the two online databases. The study of Spinola et al.[18] was focused on the association between *FGFR4 *Gly^388^Arg polymorphism and lung cancer risk, and the studies of Wang et al.[12] and Sahadevan et al.[4] were not epidemiological association studies, so they were all excluded in present study. In the four papers left, FitzGerald et al.[8] and Wang et al.[11] provided data on both African-American and Caucasian. Overall, four articles (six studies) with 2618 prostate cancer cases and 2305 controls were retrieved based on the search criteria for prostate cancer susceptibility related to the *FGFR4 *Gly^388^Arg polymorphism. Study characteristics are summarized in Table 1. All the studies used frequency-matched controls to the cases by the age, sex or ethnicity, and the distribution of genotypes in the controls was consistent with Hardy-Weinberg equilibrium in all studies. Moreover, among the four articles, three[8,10,11] mentioned the association between *FGFR4 *Gly^388^Arg polymorphism and progression of prostate cancer. The stratifications for pathological parameters of the cases in the three articles were also shown in Table 1. The cases of the three articles were all stratified by Gleason score and tumor stage. However, the classification standard of Gleason score was not uniform; thus, we only focused on the association between *FGFR4 *Gly^388^Arg polymorphism and tumor stage (advanced vs. localized). Advanced stage corresponded to T3 stage in the study of Wang et al.[11], regional/distant stage in the study of FitzGerald et al.[8], and stage C+D in the study of Ma et al.[10], respectively. And localized stage meant T2 stage in the study of Wang et al., local stage in the study of FitzGerald et al., and stage A+B in the study of Ma et al., respectively.

**Table 1:** Main characteristics of selected studies

First author	Year	Ethnicity	Cases	Controls	Case			Control			Pathological parameters of cases
						
					Gly/Gly	Gly/Arg	Arg/Arg	Gly/Gly	Gly/Arg	Arg/Arg	
Wang	2004	Caucasian	284	97	125	117	42	53	40	4	Stratified by Gleason score (7-9 vs. 5-6), pathological stage (T3 vs. T2), lymph node metastasis (positive vs. negative), and PSA recurrence (positive vs. negative)
Wang	2004	African American	45	94	37	6	2	76	18	0	
FitzGerald	2009	Caucasian	1254	1251	587	544	123	631	496	124	Stratified by Gleason score [≥7(4+3) vs.≤7(3+4)], and pathological stage (regional/distant vs. local)
FitzGerald	2009	African American	146	80	104	39	3	60	18	2	
Ho	2009	Caucasian	397	439	183	182	32	235	167	37	-
Ma	2008	Asian	492	344	163	196	133	125	152	67	Stratified by tumor stage (stage D vs. A+B+C) a, and Gleason score (8-10 vs. 2-7)

^a ^Stage A (T_1a-b_N_0_M_0_), StageB (T_1c-2_N_0_M_0_), Stage C (T_3-4_N_0_M_0_) and Stage D (T_1-4_N_1_M_0-1 _orT_1-4_N_0-1_M_1_) by the modified Whitmore-Jewett system.

### Quantitative synthesis

We observed a wide variation of Arg^388 ^allele frequencies across different ethnicities. The frequency of Arg^388 ^allele was 28.90% among Caucasian controls and 41.57% among Asian controls, which were significantly higher than that in African-American controls (11.49%, *P *< 0.01).

**Figure 1:**
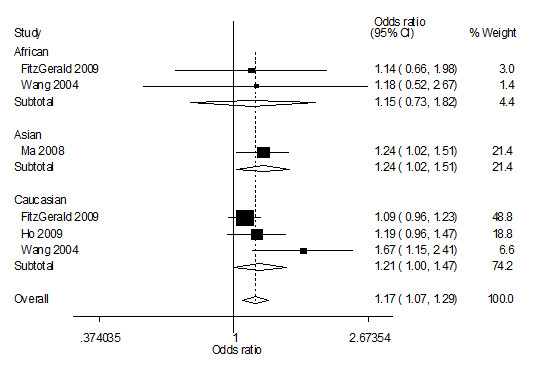
**Forest plot of prostate cancer risk associated with *FGFR4 *Gly^388^Arg polymorphism (Arg allele *vs*. Gly allele)**. The squares and horizontal lines correspond to the study-specific OR and 95% CI. The area of the squares reflects the weight (inverse of the variance). The diamond represents the summary OR and 95% CI.

Overall, the combined result based on all studies showed the evidence of an association between the increased risk of prostate cancer and the variant genotypes in different genetic models. As shown in Table 2 and Figure 1, the Arg^388 ^allele increased overall prostate cancer risk compared with Gly^388 ^allele (OR = 1.17, 95% CI = 1.07-1.29). Significant main effects were also observed in dominate genetic model (OR = 1.21, 95% CI = 1.08-1.36).

**Table 2:** Stratified analyses of the *FGFR4 *Gly^388^Arg polymorphism on cancer risk

Variables	na	Cases/controls	Arg vs. Gly		Arg/Arg vs. Gly/Gly		Gly/Arg+Arg/Arg vs. Gly/Gly		Arg/Arg vs. Gly/Gly+ Gly/Arg	
										
			OR (95% CI)	Pb	OR (95% CI)	Pb	OR (95% CI)	Pb	OR (95% CI)	Pb
Total	5	2618/2305	1.17 (1.07-1.29)	0.37	1.39 (0.97-1.99)	0.08	1.21 (1.08-1.36)	0.79	1.32 (0.90-1.94)c	0.03
Caucasian	3	1935/1787	1.21 (1.00-1.47)	0.09	1.40 (0.80-2.45)c	0.04	1.23 (1.08-1.40)	0.39	1.26 (0.72-2.19)c	0.04
African	2	191/174	1.15 (0.73-1.82)	0.95	2.17 (0.20-23.14)	0.17	1.11 (0.66-1.86)	0.62	2.21 (0.18-26.83)	0.15
Asian	1	492/344	1.24 (1.02-1.51)	-	1.52 (1.05-2.22)	-	1.15 (0.86-1.54)	-	1.53 (1.10-2.14)	-
Tumor stage										
Localized			1.00 (reference)		1.00 (reference)		1.00 (reference)		1.00 (reference)	
Advanced			1.18 (0.96-1.44)	0.75	1.33 (0.91-1.96)	0.89	1.01 (0.93-1.10)	0.44	1.34 (1.03-1.74)	0.61

^a^Number of comparisons; ^b^*P *value of *Q*-test for heterogeneity test; ^c^Random-effects model was used when *P *value for heterogeneity test < 0.05; otherwise, fixed-effects model was used;

When stratifying for race, results were similar. Specially, significantly increased risk was found among Caucasian populations (Arg^388 ^and Gly^388 ^comparison: OR = 1.21, 95% CI: 1.00-1.47; dominant genetic model: OR = 1.23, 95% CI: 1.08-1.40) and Asian population (Arg^388 ^and Gly^388 ^comparison: OR = 1.24, 95% CI: 1.02-1.51; homozygote comparison: OR = 1.52, 95% CI: 1.05-2.22; recessive genetic model: OR = 1.53, 95% CI: 1.10-2.14). Although the effect in African-Americans was in the same direction as for other groups, the difference was not statistically significant (Arg^388 ^and Gly^388 ^comparison: OR = 1.15, 95% CI: 0.73-1.82; homozygote comparison: OR = 2.17, 95% CI: 0.20-23.14; dominant genetic model: OR = 1.11, 95% CI: 0.66-1.86 and recessive genetic model: OR = 2.21, 95% CI: 0.18-26.83).

**Figure 2:**
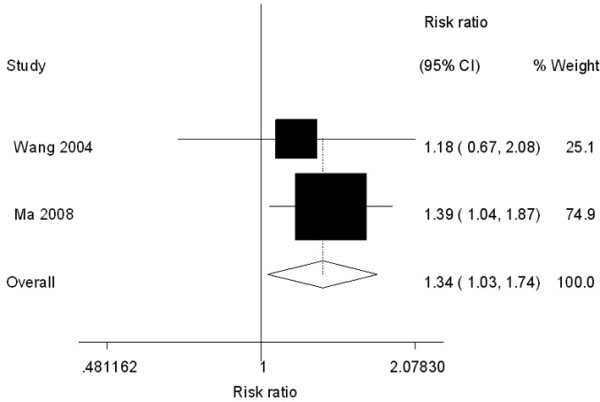
**Forest plot of prostate cancer progression associated with *FGFR4 *Gly^388^Arg polymorphism (Arg/Arg *vs*. Gly/Gly+Gly/Arg)**. The squares and horizontal lines correspond to the study-specific OR and 95% CI. The area of the squares reflects the weight (inverse of the variance). The diamond represents the summary OR and 95% CI.

In addition, when concerning tumor stage and *FGFR4 *Gly^388^Arg polymorphism, patients with prostate cancer with Arg/Arg genotype had a 1.34-fold increased risk of advanced or metastatic prostate cancer (95% CI: 1.03-1.74) compared with the Gly/Gly+Gly/Arg genotype (seen Figure 2).

### Sensitivity analysis

Sensitivity analysis was performed by sequential omission of individual studies. The pooled 95% CI for Arg^388 ^vs. Gly^388 ^was consistently over 1.0, indicating that the results of this meta-analysis are stable.

### Publication bias

**Figure 3:**
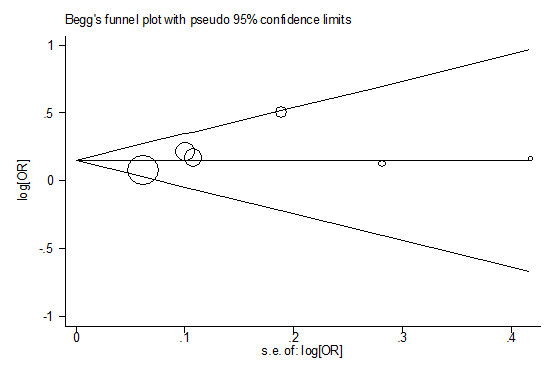
**Begg's funnel plot for publication bias test**. Each point represents a separate study for the indicated association. Log[OR], natural logarithm of OR. Horizontal line, mean effect size.

Begg's funnel plot and Egger's test were performed to assess the publication bias. The shape of the funnel plots seemed symmetrical in the comparison of the Arg^388 ^*vs*. Gly^388 ^(Figure 3). Furthermore, Egger's test was used to provide statistical evidence for funnel plot symmetry (*t *= 1.30, *P *= 0.26), suggesting that no publication bias was exist.

## Discussion

The present meta-analysis, including 2,618 cases and 2,305 controls from six published studies, explored the association between *FGFR4 *Gly^388^Arg polymorphism and development and progression of prostate cancer. To the best of our knowledge, this is the first meta-analysis to explore *FGFR4 *Gly^388^Arg polymorphism in development and progression of prostate cancer. The results indicated that *FGFR4 *Arg^388 ^allele is a potential risk factor for developing and progressing prostate cancer. These findings may be biologically plausible. The *FGFR4 *Gly^388^Arg polymorphism results in an amino acid change in the transmembrane domain of the receptor, which may alter the activity of the receptor. FGFR4 is the activator of the MAPK signaling cascade, yet it is a principal receptor for key mitogenic FGFs in prostate cancer cells[19-21]. There was evidence that FGFR4 contributed to progression in liver, lung, colon tumors[22] and prostate cancer[12]. The effects of *FGFR4 *Arg^388 ^allele may also predispose cancer patients to disease progression, based on the reported significant association between *FGFR4 *genotype and tumor aggressiveness (lymph node involvement, advanced stage) or patients' survival[6,7], and the results about its biological role on cancer cell motility and invasiveness[11]. In our meta-analysis, we found that subjects carrying Arg^388 ^were associated with higher risk of developing and progressing prostate cancer than those with the wild-type allele, which confirmed the hypothesis described above.

Some limitations of this meta-analysis should be acknowledged. First of all, the control populations were not uniform. Healthy populations as well as non-cancer patients like BPH patients were included. Some individuals in the control group are likely to develop cancer in subsequent years though they had no clinical symptoms at the time of investigation. Misclassification bias results in deviation of genotype distribution in the controls. Second, prostate cancer, as a complex disease, was considered as the result of combined effects of multi-factors, including inherited and environmental factors[23], however, no such data was observed in previous studies. Thus, our result was only based on unadjusted estimates. Lacking of the information for the data analysis may cause serious confounding bias. Third, the effect of the polymorphism was relatively trivial with small ORs. We need further studies with larger number participants to confirm the effect.

Our meta-analysis also had some advantages. First, disease progression status as tumor stage was taken into account in present study. Second, data in present study were pooled from different studies, which significantly increased statistical power of the analysis. Third, the quality of studies included in our meta-analysis was satisfactory and cruelly met our inclusion criterion. Fourth, the distribution of genotypes in the controls was consistent with Hardy-Weinberg equilibrium in all studies. We further performed sensitivity analysis to detect the stability of the meta-analysis, and the results did not alter the pattern of association and revealed that the risk effect of Arg^388 ^was stable. In addition, publication bias was not detected in present study, indicating that our findings seemed not to be due to biased publications.

## Conclusions

Our meta-analysis showed the evidence that *FGFR4 *Arg^388 ^allele was associated with an increased risk of prostate cancer development and progression, suggesting that *FGFR4 *Gly^388^Arg polymorphism could be a marker for prostate cancer development and progression. Based on the limitations of present study list above, further prospective researches using standardized unbiased methods, and larger numbers of worldwide participants are expected to examine the association to confirm our results, and other possible confounding risk factors like age, life style, and familial history should also be controlled when it was assessed. Moreover, gene-gene and gene-environment interactions should also be considered.

## Abbreviations

FGFR4: Fibroblast growth factor receptor 4; Gly: glycine; Arg: arginine; OR: odds ratio; CI: confidence interval.

## Competing interests

The authors declare that they have no competing interests.

## Authors' contributions

BX participated in study design and drafted the manuscript.

SQC participated in collection of data and manuscript preparation.

NT and ZDZ performed the statistical analysis and participated in the critical revision of the manuscript.

ZJW critically revised the manuscript.

MC and LXH participated in its design.

All authors read and approved the final manuscript.

## Pre-publication history

The pre-publication history for this paper can be accessed here:

http://www.biomedcentral.com/1471-2407/11/84/prepub
